# Asymmetric Osmoadaptive Responses in Intermediate-Salinity Microbial Communities Revealed by Metatranscriptomics

**DOI:** 10.3390/ijms27115114

**Published:** 2026-06-05

**Authors:** Salvador Mirete, María Lamprecht-Grandío, Carolina González de Figueras, José Eduardo González-Pastor

**Affiliations:** Centro de Astrobiología (CAB), CSIC-INTA, Carretera de Ajalvir km 4, Torrejón de Ardoz, 28850 Madrid, Spain; maria.lamprecht@pdi.atlanticomedio.es (M.L.-G.); gonzalezfc@cab.inta-csic.es (C.G.d.F.);

**Keywords:** hypersaline ecosystem, extremophiles, proteome, Pseudomonadota, Halobacteriota, Bacteroidota

## Abstract

Salinity is a dominant ecological driver shaping microbial community structure and function in hypersaline environments. Here, we investigated transcriptional responses to rapid salinity fluctuations using metatranscriptomic analyses of an intermediate-salinity brine sample from the Santa Pola solar salterns (Alicante, Spain). To this end, two experimental conditions were applied: salinity increase (12.4% to 17%) and salinity dilution (12.4% to 7%). Differential gene expression, functional enrichment, and protein isoelectric point (pI) distributions were analyzed to characterize osmoadaptive mechanisms. Salinity increase triggered a stress-dominated response characterized by upregulation of compatible solute biosynthesis (e.g., glycine betaine and ectoine), protein turnover, and chaperone activity, alongside repression of translation, energy metabolism, and transport systems. In contrast, salinity dilution induced metabolic reactivation, including enhanced translation, energy production, and osmolyte degradation pathways, indicating recovery from osmotic stress. Functional shifts were accompanied by changes in proteome physicochemical properties, with increased salinity promoting a shift toward higher pI proteins, consistent with salt-out strategies. These findings reveal a highly dynamic and asymmetric transcriptional plasticity, where osmotic upshift imposes stronger constraints than downshift, driving coordinated metabolic reprogramming and proteome restructuring in intermediate-salinity microbial communities.

## 1. Introduction

Salinity is a key environmental driver shaping microbial distribution and diversity across most of Earth’s aquatic systems, particularly in marine-derived hypersaline habitats [1]. In systems such as the Santa Pola solar salterns (Alicante, Spain), stable salinity gradients from seawater to halite saturation provide a natural model to study microbial adaptation under extreme osmotic stress conditions [2]. These gradients generate sharp physicochemical transitions over relatively short spatial scales, allowing the coexistence of taxonomically and functionally distinct microbial communities [2]. Such conditions impose strong selective pressures that act not only on community composition but also on metabolic activity and regulatory responses. As a result, these salterns represent ideal environments to investigate how microorganisms fine-tune their physiological strategies in response to changing salinity, where we previously studied adaptation mechanisms at the transcriptional level in response to both high-salt and dilution stresses [3].

Despite the wealth of taxonomic data, a profound knowledge gap remains regarding the molecular mechanisms by which microbial communities actively respond to short-term, dynamic environmental perturbations. Salinity in solar salterns is not a constant parameter [4]; it is subject to rapid shifts caused by external events, such as heavy rainfall inducing sudden dilution or intense solar radiation causing rapid salt concentration [5]. It is established that such osmotic shocks can reduce biodiversity or alter microbial abundance, and the genome-level functional shifts and transcriptional mediation that facilitate these transitions were previously characterized on high-salinity regimes (20–30%) [3]. Nevertheless, this deficiency is particularly acute in intermediate-salinity habitats, where the community must navigate the threshold between better studied moderate and extreme salt concentrations.

In the present study, we explored transcriptional plasticity under contrasting salinity perturbations from the Santa Pola salterns starting from 12.4%. We set two experiments mimicking salinity concentration by evaporation (12.4% to 17%) and salinity dilution by rainfall (12.4% to 7%). We characterized expressed genes through the Clusters of Orthologous Groups (COG) [6] and Gene Ontology (GO) [7] frameworks. We hypothesized that sudden salinity shifts trigger distinct transcriptional adjustments with functional resilience.

## 2. Results and Discussion

### 2.1. Community Structure

**Figure 1:**
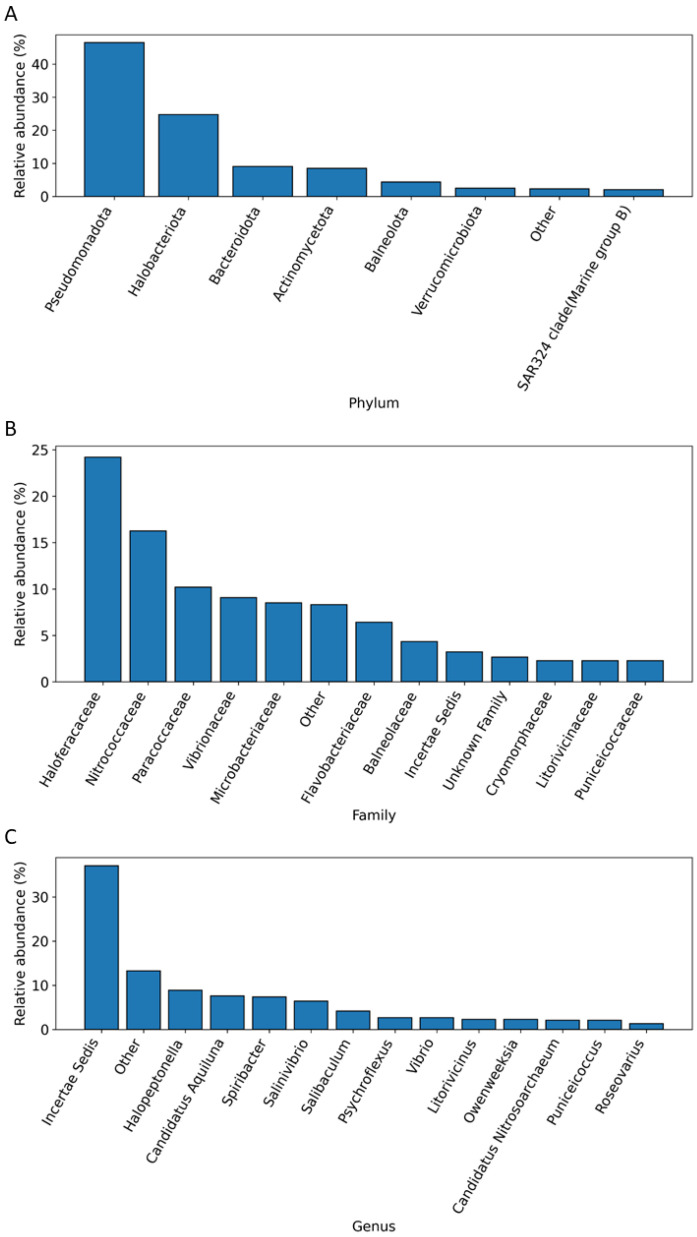
Taxonomic composition of the BRAS intermediate-salinity microbial community based on 16S rRNA gene sequences retrieved from the metagenomic dataset. The BRAS sample was collected from an intermediate-salinity pond (12.4% total salts) of the Santa Pola solar salterns (Alicante, Spain). Bar plots show the relative abundance (%) of microbial taxa at the (**A**) phylum, (**B**) family, and (**C**) genus levels. Only taxa contributing ≥1% of total abundance at each taxonomic rank are shown individually, whereas low-abundance (<1%) taxa were grouped into the “Other” category. The community was dominated by members of Pseudomonadota, together with representatives of Halobacteriota and Bacteroidota, consistent with the taxonomic structure characteristic of intermediate hypersaline environments.

**Figure 2:**
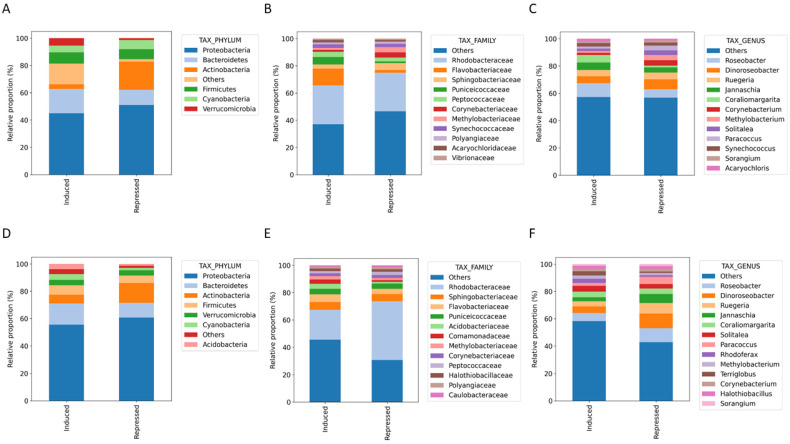
Taxonomic distribution of expressed genes in metatranscriptomic datasets obtained after experimental salinity perturbations. The BRAS microbial community (initial salinity 12.4%) was subjected to two osmotic shock experiments: salinity concentration to 17% (BRAS3) and salinity dilution to 7% (BRAS2). Bar plots show the relative abundance (%) of taxonomically assigned expressed genes at the phylum, family, and genus levels for induced and repressed transcripts in each experiment: (**A**–**C**) BRAS3 and (**D**–**F**) BRAS2. Only taxa representing >1% abundance at the phylum level or >10% abundance at the genus level in at least one dataset are shown individually. Taxonomic profiles remained broadly similar across treatments, although differences in transcriptional activity were observed among several dominant microbial groups.

Taxonomic profiling revealed a highly uneven microbial community dominated by a limited number of lineages and a long tail of low-abundance taxa (Figure 1 and Figure 2). The presence of a broad diversity of low-abundance lineages, aggregated in the “Others” category, suggests the presence of a microbial seed bank that maintains functional potential across fluctuating environmental conditions [8].

At the phylum level, Pseudomonadota (formerly Proteobacteria) dominated both the 16S rRNA and metatranscriptomic profiles, with increased transcriptional representation under repressed states in both perturbations (Figure 2). This pattern is consistent with intermediate-salinity systems where members of this phylum often dominate community [9]. In addition, Halobacteriota (formerly Euryarchaeota) were abundant in the 16S rRNA dataset but underrepresented in the metatranscriptome, suggesting lower transcriptional activity under the tested conditions and reinforcing the interpretation of a sub-saturated hypersaline environment [9]. Bacteroidota showed increased transcriptional activity under both perturbations, suggesting higher functional resilience, whereas Actinobacteria displayed reduced transcriptional responsiveness, indicating sensitivity to osmotic shifts.

At finer taxonomic resolution, *Rhodobacteraceae* showed increased repression under the dilution condition, while *Flavobacteriaceae* were preferentially induced under salinity increase, indicating differential sensitivity among dominant lineages. At the genus level, *Roseobacter*-related taxa exhibited contrasting expression patterns between dilution and concentration treatments, reflecting high metabolic plasticity. The detection of *Spiribacter* (Figure 1C) further supports the relevance of salt-out strategies, which are typically associated with intermediate salinity regimes [10,11].

### 2.2. Functional Reorganization of the Hypersaline Microbial Community Under Opposing Salinity Shifts

To our knowledge, no additional metatranscriptomic studies have been conducted in this environment apart from our own previous work [3]. That earlier study was performed on samples collected within the same area; however, it represented a distinct, more hypersaline environment characterized by markedly different microbial diversity and adaptive response mechanisms. This distinction is particularly relevant for the interpretation of the present results, as the functional and transcriptional patterns observed here likely reflect ecological adaptations specific to the lower-salinity conditions analyzed in this study, rather than general responses shared across all hypersaline niches.

**Figure 3:**
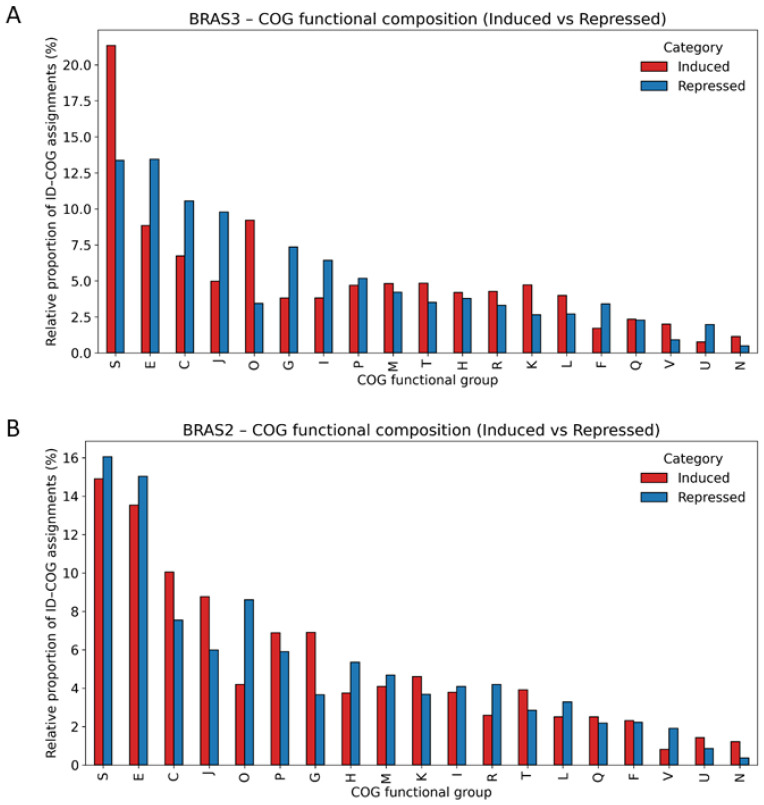
Functional organization of differentially expressed genes based on COG categories in response to salinity perturbations. The BRAS microbial community was exposed to either increased salinity (BRAS3; 12.4% to 17%) or reduced salinity (BRAS2; 12.4% to 7%), and differentially expressed genes were classified as induced (FoldChange > 2) or repressed (FoldChange < −2). Panels show the relative contribution of COG functional groups in (**A**) BRAS3 and (**B**) BRAS2. Only categories contributing >1% in at least one condition are displayed. Salinity increase promoted transcriptional investment in stress-response and protein turnover functions (Category O), whereas salinity dilution enhanced translation and energy-production categories (J and C), indicating contrasting physiological states under osmotic upshift and downshift conditions. COG functional categories are defined as follows: C, energy production and conversion; D, cell cycle control, cell division and chromosome partitioning; E, amino acid transport and metabolism; F, nucleotide transport and metabolism; G, carbohydrate transport and metabolism; H, coenzyme transport and metabolism; I, lipid transport and metabolism; J, translation, ribosomal structure and biogenesis; K, transcription; L, replication, recombination and repair; M, cell wall/membrane/envelope biogenesis; N, cell motility; O, posttranslational modification, protein turnover and chaperones; P, inorganic ion transport and metabolism; Q, secondary metabolites biosynthesis, transport and catabolism; R, general function prediction only; S, function unknown; T, signal transduction mechanisms; U, intracellular trafficking, secretion and vesicular transport; V, defense mechanisms.

In this study we observed that COG-based metatranscriptomic profiling revealed a conserved functional structure with marked differences between induced and repressed genes depending on the direction of the salinity shift (Figure 3). While the community maintained a conserved functional hierarchy dominated by a narrow set of core categories, a remarkable difference occurred between the induced and repressed states in the most abundant COGs (e.g., S, E, C, J, O, P and G; see Figure 3 legend for category letter codes) depending on the salt shock. Categories S and E were the most represented across conditions, indicating that a substantial fraction of transcriptional activity is associated with poorly characterized functions and amino acid metabolism.

Salinity increase (BRAS3) resulted in reduced expression of Categories C and J, alongside increased representation of Category O, consistent with stress-response activation and in line with our previous metatranscriptomic survey [3]. This pattern indicates a shift toward stress-response processes and reduced biosynthetic activity, consistent with a transition to a maintenance-oriented physiological state under osmotic stress.

In contrast, salinity dilution (BRAS2) promoted increased expression of Categories C and J, coupled with reduced representation of Category O, indicating a shift toward growth-associated processes. This coordinated shift suggests a transition toward growth-associated processes following osmotic relaxation. Together, these results highlight a clear functional asymmetry, with osmotic upshift imposing stronger constraints on cellular activity than dilution. 

It is important to note that metatranscriptomic profiles at the community level reflect both transcriptional regulation within individual taxa and changes in the relative activity or contribution of different community members. As such, the observed functional shifts cannot be unequivocally attributed to coordinated regulatory responses across the entire community but may instead arise from differential activation of specific populations. Therefore, interpretations of community-wide responses should be considered with caution.

### 2.3. Osmoadaptation Strategies: Synthesis Versus Turnover of Compatible Solutes

Functional enrichment analysis further supports this asymmetric response.

**Figure 4:**
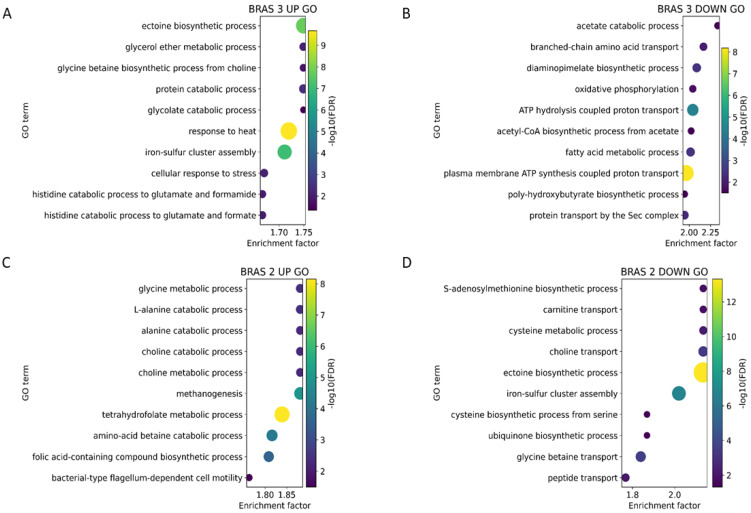
Functional enrichment analysis of GO Biological Process (GO:BP) terms associated with differential gene expression under salinity perturbation experiments. The BRAS microbial community was experimentally exposed to salinity concentration (BRAS3; 12.4% to 17%) and salinity dilution (BRAS2; 12.4% to 7%). Panels show the top 10 significantly enriched GO:BP terms for (**A**) upregulated genes in BRAS3, (**B**) downregulated genes in BRAS3, (**C**) upregulated genes in BRAS2, and (**D**) downregulated genes in BRAS2. Enrichment analyses were performed using Fisher’s exact test followed by Benjamini–Hochberg false discovery rate correction (FDR < 0.05). Increased salinity induced pathways associated with compatible solute biosynthesis, stress response, and protein turnover, whereas salinity dilution stimulated osmolyte catabolism, amino-acid metabolism and one-carbon metabolism, together with growth-associated functions including translation and motility-related processes.

Under increased salinity (BRAS3), compatible solute biosynthesis pathways, particularly glycine betaine and ectoine, were significantly enriched (Figure 4A and Table S4), indicating activation of osmoprotective mechanisms. These results are in agreement with the presence of several genes involved in compatible solute biosynthesis retrieved from the intermediate salinity Santa Pola ponds (SS13, 13% salinity; SS19, 19% salinity) [9], and in the genomes of *Spiribacter* [10] and members of the *Rhodobacteraceae* [12]. Concurrent enrichment of stress-response processes and chaperone-related functions (e.g., DnaK) suggests increased demand for protein stabilization under hyperosmotic conditions.

In contrast, salinity dilution experiment (BRAS2) was characterized by enrichment of osmolyte degradation pathways, including choline and glycine betaine catabolism, indicating recycling of previously accumulated compatible solutes (Figure 4C and Table S4). Concomitantly, osmoprotectant biosynthesis and transport systems (e.g., ectoine synthesis and compatible solute transporters) were significantly repressed, consistent with reduced osmotic stress and decreased demand for osmolyte accumulation (Figure 4D and Table S4). 

These opposing patterns reflect a dynamic regulation of osmolyte pools, with synthesis dominating under osmotic upshift and turnover prevailing under dilution.

### 2.4. Global Functional Responses Associated with Salinity Perturbations

Beyond osmoadaptation, salinity perturbations induced broader metabolic adjustments. Under increased salinity (BRAS3), enrichment of protein catabolism and iron–sulfur cluster assembly suggests enhanced protein turnover and adjustments in redox metabolism (Figure 4A and Table S4). These changes were accompanied by repression of translation and transport-related functions, including ribosomal proteins and nutrient uptake systems, consistent with reduced biosynthetic activity and a shift toward maintenance-dominated metabolism (Figure 4B and Table S4). 

In contrast, salinity dilution (BRAS2) promoted upregulation of translation and metabolic pathways associated with amino acid turnover and one-carbon metabolism, reflecting increased biosynthetic activity and intracellular recycling (Figure 4C and Table S4). Motility-related functions were also upregulated, suggesting enhanced environmental exploration under reduced salinity [8]. Concurrent downregulation of stress-associated pathways such as the iron–sulfur cluster assembly (Figure 4D and Table S4), further supports the interpretation of reduced physiological constraints [13]. 

### 2.5. Protein Isoelectric Point Distributions in Salinity Perturbation Experiments

**Figure 5:**
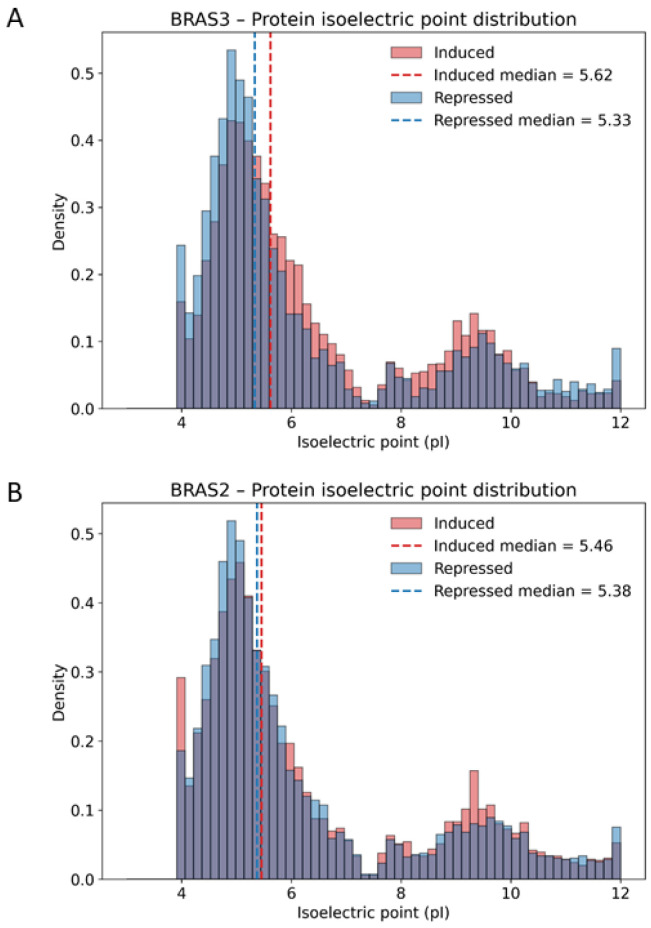
Distribution of predicted pI values in differentially expressed genes under contrasting salinity perturbations. Predicted protein sequences derived from metatranscriptomic datasets were analyzed for theoretical pI values in (**A**) BRAS3 (salinity concentration from 12.4% to 17%) and (**B**) BRAS2 (salinity dilution from 12.4% to 7%). Histograms compare pI distributions between proteins encoded by induced genes (FoldChange > 2) and repressed genes (FoldChange < −2). Vertical dashed lines indicate median pI values for each protein set. The BRAS3 experiment showed a significant shift toward higher pI values among induced proteins, suggesting proteome restructuring associated with salt-out osmoadaptation. In contrast, BRAS2 displayed only minor changes in pI distributions, indicating a comparatively weaker proteome-level response following osmotic relaxation.

Analysis of pI distributions revealed consistent bimodal patterns across both experiments, with a dominant acidic peak (pI 4.5–6.0) and a secondary basic peak (pI 9–10), typical of halophilic proteomes (Figure 5) [14,15].

Under salinity increase (BRAS3), induced proteins showed a shift toward higher pI values compared to repressed proteins (median Δ = 0.29), with increased representation of intermediate and basic pI ranges. These differences were statistically significant, indicating a non-random redistribution of protein charge properties (Table S5). However, given the community-level nature of the analysis and the short timescale, this pattern likely reflects shifts in the relative contribution of functionally distinct populations rather than large-scale proteome restructuring.

In contrast, salinity dilution (BRAS2) showed only minor differences between induced and repressed proteins (Δ = 0.08), with largely overlapping distributions. Although statistically significant, the small effect size suggests limited biological impact.

Taken together, these results support an asymmetric physicochemical response, with more pronounced shifts under osmotic upshift. This pattern is consistent with the predominance of salt-out strategies in intermediate salinity systems and highlights the stronger constraints imposed by increasing salinity on microbial community function [16].

## 3. Materials and Methods

### 3.1. Sample and Experiment Description

Water samples were collected on 7 September 2016, from an evaporation pond within the Santa Pola solar saltern system (Alicante, eastern Spain; GPS coordinates 38°11′45.3″ N 0°35′52.7″ W). This multi-pond saltern system consists of a series of shallow ponds in which seawater evaporates progressively, producing a salinity gradient that ranges from marine levels to near saturation. A salinity environmental sample (BRAS) was recovered from an intermediate-salinity pond (12.4% salinity), which along with other samples analysed in a previous work [3], reflect the natural range of salt concentrations found within the saltern system.

The salinity was determined using a hand refractometer (Hanna Instruments, Eibar, Spain). A total of 1 L of the BRAS sample was collected using sterile plastic jars.

The concentration experiment (BRAS3) was aimed to induce the expression of genes involved in adaptation to elevated salinity. The BRAS sample with an initial salinity of 12.4% (filtered through sterile cloth using a stainless-steel strainer to remove particulates) was concentrated to a final salinity of 17% by addition of a SW 30% solution (30% NaCl solution), resulting in a final volume of 160 mL. Control samples received the same volume of SW 12.4% to maintain constant salinity conditions. Samples were incubated for 1 h, and three aliquots of 50 mL were subsequently collected. Cells were recovered by centrifugation for 20 min at 10,000 rpm and 4 °C, pooled into two Eppendorf tubes (Sarstedt, Nümbrecht, Germany), centrifuged again at 10,000 rpm for 4 min, and rapidly frozen in dry ice.

The dilution experiment (BRAS2) was designed to induce the transcription of genes associated with adaptation to reduced salinity conditions. The BRAS sample with an initial salinity of 12.4% (filtered through sterile cloth using a stainless-steel strainer to remove particulates) was diluted to 7% salinity using Milli-Q water, reaching a final volume of 330 mL. Control treatments were prepared by adding the same volume of a SW 12.4% solution (12.4% NaCl solution) in order to maintain a constant salinity. All samples were incubated for 1 h, after which three aliquots of 50 mL were collected. Cells were pelleted by centrifugation for 20 min at 10,000 rpm and 4 °C, pooled into two Eppendorf tubes, centrifuged again at 10,000 rpm for 4 min, and immediately frozen in dry ice.

Each experiment was conducted with three independent biological replicates which correspond to independent experimental treatments derived from the same initial environmental sample, and therefore do not capture natural spatial or temporal variability of the ecosystem.

### 3.2. Nucleic Acid Extraction, Sequencing, and Bioinformatic Analyses

Microbial biomass was collected by filtration (0.22 µm), and DNA/RNA were extracted using CTAB-based protocols [17] and commercial kits, followed by quality assessment and Illumina HiSeq 2500 (Illumina, Inc., San Diego, CA, USA) paired-end sequencing (DNA and rRNA-depleted RNA). Libraries were prepared using standard TruSeq workflows, and sequencing was performed at Sistemas Genómicos S.L. (Valencia, Spain).

DNA sequencing from the BRAS sample yielded 109.9 million reads. RNA sequencing yielded between 30.8 and 23.4 for BRAS2 and between 31.4 and 23.3 for BRAS3 (Table S3). Consistency across biological replicates (Figures S1 and S2) was used as an additional quality control criterion.

Raw reads were quality-filtered, trimmed, and assembled using Megahit v1.0 [18]. Gene prediction was accomplished with Glimmer3 [19] and taxonomic/functional annotation was based on curated databases (e.g., UniProt, KEGG, SILVA). Transcriptomic reads were mapped to assemblies, quantified, and statistically analyzed with DESeq2 [20] to identify differentially expressed genes (FDR ≤ 0.05; |FC| ≥ 2) (Tables S1 and S2). Due to the absence of a reference genome for the hypersaline sample studied, the metatranscriptomic reads from each sample were aligned against a metagenome assembled from the same sample to identify differentially expressed genes. Only reads that successfully matched in silico between the metatranscriptome and the corresponding metagenome were considered.

Differential expression analyses were performed using time-matched untreated controls (1 h incubation) to account for background transcriptional changes unrelated to salinity perturbation.

Functional enrichment analyses were conducted on differentially expressed genes from the BRAS2 and BRAS3 experiments using a custom Python workflow. Genes were classified as up-regulated or down-regulated based on fold-change thresholds (Fold-Change > 2 for up-regulation and <−2 for down-regulation). Functional annotations were derived from COG [6] and GO [7] terms assigned at the gene level. When multiple COG annotations were associated with a single gene, each annotation was considered independently in the enrichment analysis. For GO enrichment, only Biological Process (BP) terms were retained, based on GO annotations prefixed as biological processes. Due to the many-to-many relationship between genes and functional annotations, individual genes could contribute to multiple functional categories. Therefore, enrichment results were interpreted as indicative of functional trends rather than strictly independent statistical events.

Enrichment for each functional category was evaluated using Fisher’s exact test (one-tailed, alternative = “greater”), comparing the number of genes associated with a given term in each regulated gene set against its frequency in the corresponding background gene population. Enrichment factors were calculated as the ratio between observed and expected proportions of genes assigned to each functional category. Resulting *p*-values were corrected for multiple testing using the Benjamini–Hochberg procedure false discovery rate (FDR) procedure. Functional terms with FDR < 0.05 were considered significantly enriched.

Detailed protocols, parameters, and computational workflows are found in our previous report [3].

### 3.3. Analysis of Protein Isoelectric Point Distributions

Protein sequences corresponding to differentially expressed genes were obtained from the metatranscriptomic datasets generated in the BRAS2 (salinity dilution) and BRAS3 (salinity concentration) experiments. Each sequence was associated with its corresponding FoldChange value (FC > 2, induced; FC < −2, repressed) derived from the differential expression analysis.

The theoretical pI of each protein was calculated from its amino acid sequence using the IsoelectricPoint module implemented in Biopython 1.84. The distribution of pI values was visualized using normalized histograms. Differences between the pI distributions of induced and repressed proteins were evaluated using two complementary non-parametric statistical tests. The Kolmogorov–Smirnov test was used to assess differences between the overall distributions, whereas the Mann–Whitney U test was used to evaluate differences in central tendency between groups.

All analyses were performed in Python 3.12.2, using pandas for data handling, NumPy 1.26.4 for numerical calculations, Matplotlib 3.9.0 for visualization, and Biopython for protein physicochemical property calculations.

## 4. Conclusions

**Figure 6:**
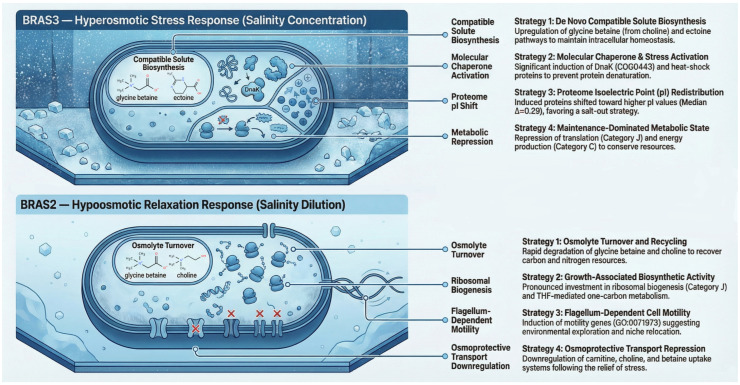
Conceptual model summarizing metatranscriptomic responses of the BRAS intermediate-salinity microbial community to abrupt salinity perturbations. The microbial assemblage from the Santa Pola solar salterns was exposed to two experimental conditions: salinity concentration (BRAS3; 12.4% to 17%) and salinity dilution (BRAS2; 12.4% to 7%). Increased salinity induced a stress-dominated physiological state characterized by activation of compatible solute biosynthesis, chaperone systems, protein turnover, and repression of growth-associated functions such as translation and energy metabolism. In contrast, salinity dilution promoted metabolic reactivation, osmolyte degradation, increased translation, and motility-related functions. The model highlights the asymmetric transcriptional plasticity and functional buffering mechanisms underlying microbial adaptation to rapid osmotic fluctuations in this intermediate hypersaline environment.

The results presented here reveal that microbial communities inhabiting intermediate hypersaline environments respond to abrupt salinity perturbations through rapid and finely tuned transcriptional reprogramming. The contrasting responses observed between salinity increase and dilution highlight a clear functional asymmetry: osmotic upshift drives a constrained, stress-dominated state characterized by enhanced protein repair/stabilization, osmolyte biosynthesis, and reduced biosynthetic activity, whereas osmotic downshift promotes metabolic reactivation, increased translation, bacterial motility and active turnover of previously accumulated compatible solutes (Figure 6).

Moreover, increasing salinity likely induces measurable changes in protein physicochemical properties, whereas decreasing salinity results in comparatively minor adjustments. This suggests that osmotic upshift imposes stronger constraints on cellular systems, requiring coordinated transcriptional and proteomic responses. Collectively, these results point to that salinity-driven gene expression changes are accompanied by shifts in protein charge properties, particularly under increasing salinity, and are consistent with the predominance of salt-out strategies in shaping the observed patterns.

The present dataset provides a valuable baseline for future comparative and longitudinal studies evaluating microbial responses to environmental variability and climate-driven change in hypersaline systems. Integrating previous and newly generated metatranscriptomic data from the same environment may help distinguish transient from persistent functional responses and clarify the relative contributions of short-term physiological plasticity and longer-term ecological dynamics under fluctuating salinity regimes. Future temporal analyses may also determine whether the constraints imposed by osmotic upshift represent a conserved ecological pattern.

## Data Availability

Sequence reads generated in the present study were deposited in the NCBI SRA under the Bioproject accession number PRJNA1426145.

## Supplementary Materials

The following supporting information can be downloaded at: https://www.mdpi.com/article/10.3390/ijms27115114/s1.
