# Supplementary material for: Asymmetric Osmoadaptive Responses in Intermediate-Salinity Microbial Communities Revealed by Metatranscriptomics
Source: Int J Mol Sci. 2026 Jun 5;27(11):5114. doi: 10.3390/ijms27115114 (PMC13257453; doi:10.3390/ijms27115114)
Supplement: Supplementary file 1 [file ijms-27-05114-s001.zip › Table S3.pdf]

**METAGENOME**		
Total sequenced (Gpb)	11	
Conting number	47784	
Mean sizes of the contigs	2494.77	
Contings range size	1000-128354	
Gene number	140148	
**METATRANSCRIPTOME**	**BRAS2**	**BRAS3**
Read counts (min–max)	23487554–30815030	23372618–31420396
Mean reads	26613908	29061078
Total sequenced genes	9363	12390
Total differential expressed genes	8001	10075
Annotated Induced genes (>2)	4335	5977
Annotated Repressed genes (<2)	3666	4098

Table S3. Metagenomics assembly and metatranscriptomic sequences summary for sample BRAS.
