# Supplementary figures and images for: Asymmetric Osmoadaptive Responses in Intermediate-Salinity Microbial Communities Revealed by Metatranscriptomics
Source: Int J Mol Sci. 2026 Jun 5;27(11):5114. doi: 10.3390/ijms27115114 (PMC13257453; doi:10.3390/ijms27115114)

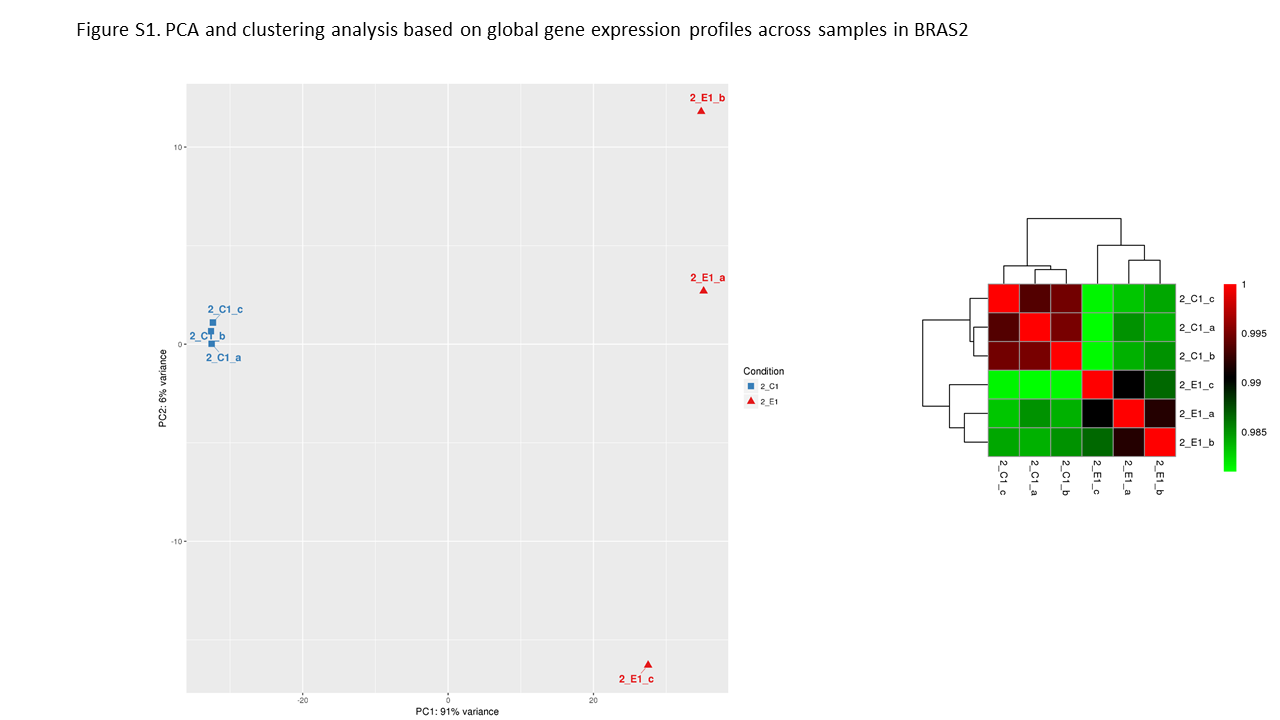

Supplement: Supplementary file 1 [file ijms-27-05114-s001.zip › Figure S1.PNG]

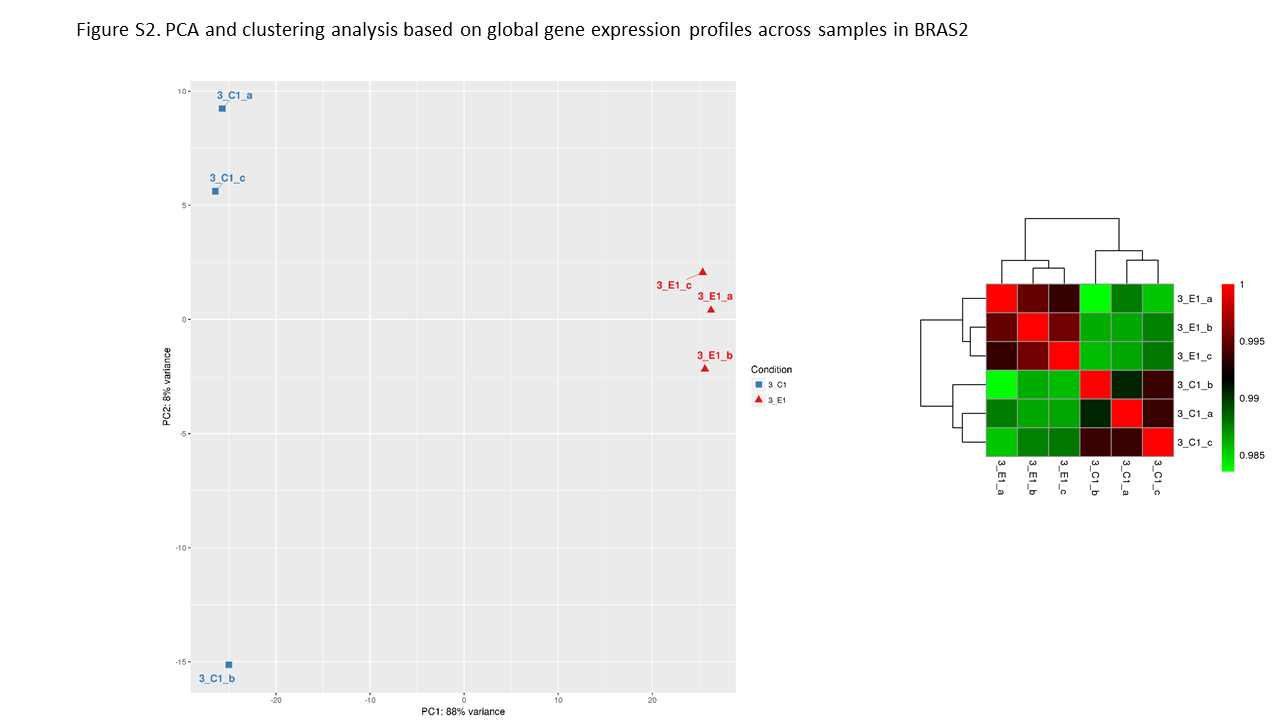

Supplement: Supplementary file 1 [file ijms-27-05114-s001.zip › Figure S2.PNG]
